# Temporal Trends in the Incidence and Disability Adjusted Life Years of Schizophrenia in China Over 30 Years

**DOI:** 10.3389/fpsyt.2022.831188

**Published:** 2022-03-09

**Authors:** Wanyue Dong, Yunning Liu, Jianzhong Sun, Yan Liu, Zhonghe Sun, Ruhai Bai

**Affiliations:** ^1^School of Elderly Care Services and Management, Nanjing University of Chinese Medicine, Nanjing, China; ^2^National Center for Chronic and Noncommunicable Disease Control and Prevention, Chinese Center for Disease Control and Prevention, Beijing, China; ^3^Health Science Center, Xi’an Jiaotong University, Xi’an, China; ^4^School of Public Administration, Huazhong Agricultural University, Wuhan, China; ^5^Department of Social Work, Nanjing First Hospital, Nanjing Medical University, Nanjing, China; ^6^School of Public Affairs, Nanjing University of Science and Technology, Nanjing, China

**Keywords:** age-period-cohort analysis, schizophrenia, incidence, trends, China, disability adjusted life years (DALYs)

## Abstract

**Background:**

Schizophrenia is an important public health problem in China. This study aims to assess the long-term trends in the incidence and disability-adjusted life years (DALYs) rate of schizophrenia in China between 1990 and 2019.

**Methods:**

The incidence and DALYs data were drawn from the Global Burden of Disease Study 2019, and an age–period–cohort model was used in the analysis.

**Results:**

The age-standardized incidence rate (ASIR) and age-standardized DALYs rate (ASDR) of schizophrenia increased by 0.3 and 3.7% for both sexes between 1990 and 2019. For males, the local drift for incidence was higher than 0 (*P* < 0.05) in those aged 10 to 29 years (local drifts, 0.01 to 0.26%) and lower than 0 (*P* < 0.05) in those aged 35 to 74 years (local drifts, −1.01 to −0.06%). For females, the local drift was higher than 0 (*P* < 0.05) in those aged 10 to 34 years (local drifts, 0.05 to 0.26%) and lower than 0 (*P* < 0.05) in those aged 40 to 74 years (local drifts, −0.86 to −0.11%). The local drift for DALYs rate was higher than 0 (*P* < 0.05) in the age group from 10 to 69 years (local drifts, 0.06 to 0.26% for males and 0.06 to 0.28% for females). The estimated period and cohort relative risks (RR) for DALYs rate of schizophrenia were found in monotonic upward patterns, and the cohort RR for the incidence increased as the birth cohort moved forward starting with those born in 1972.

**Conclusion:**

Although the crude incidence of schizophrenia has decreased in China, the ASIR, ASDR, and crude DALYs rate all showed a general increasing trend over the last three decades. The DALYs rate continue to increase as the birth cohort moved forward, and the increasing trend of incidence was also found in individuals born after 1972. More efforts are needed to promote mental health in China.

## Introduction

Schizophrenia is a chronic and serious mental disease characterized by distortions in language, perception, emotions, thinking, behavior and sense of self (1). Patients with schizophrenia are more likely to suffer from HIV infection, obstetric complications, cardiovascular disease, obesity and other physical diseases than the general population (2). Compared with the general population, the life expectancy of patients with schizophrenia is shortened by 10 to 25 years (3–5). Schizophrenia not only seriously affects the patients themselves and their families but also brings a heavy burden to society (6). In 2017, there were 19.78 million schizophrenic patients worldwide, an increase of 62.74% over 1990 (7), and this number may continue to rise in the future (8).

Globally, schizophrenia is more common in East Asia and South Asia, especially in China and India (7). In 2017, disability-adjusted life years (DALYs) in China and India accounted for 35% of the total DALYs of schizophrenia, with new cases accounting for 45% (7). China had the largest absolute number of newly diagnosed schizophrenia cases among the 20 most populous countries in the world in 2017 (0.29 million, 95% UI = 0.26 to 0.33 million), and it is also the country with the highest DALYs (3.67 million, 95% UI = 2.75 to 4.5 million) (7). Schizophrenia has become an important public health problem in China (9).

In 2008, China enacted the “*National Guiding Outline for the Development of the Mental Health Service System* (2008–2015), followed by regulations and laws including “*Regulations on the Management of Severe Mental Diseases (2012 Edition)*” and “*Mental Health Law.*” These efforts all contributed to promoting the development of mental health in China (10). From 2005 to 2015, the number of specialist psychiatric hospitals nationwide was 557 in 2005, 874 in 2010, and 1235 in 2015. Meanwhile, the total number of psychiatric beds increased from 109,000 in 2005 to 433,100 in 2015. In 2015, the number of psychiatric beds per 10,000 populations nationwide was 3.15, and the number of psychiatric professionals per 10,000 populations was 4.16 (10). However, there is still gap when compared with the average number of psychiatric beds and psychiatric professionals in middle and high-income countries (10). Moreover, 29% of registered psychiatrists in China only had a junior college diploma, and 14% had not received any training (10).

Previous studies have shown the trend in the prevalence of schizophrenia in China over time (11). However, few studies have explored changes in the incidence and DALYs rates of schizophrenia in different age groups in China. Furthermore, the potential effects underlying the temporal trends are still unknown. Statistical methods used commonly to assess the trends of disease in populations are descriptive, agnostic, and non-parametric (12), which may miss some important information. Parametric statistical models would play a more prominent role, especially the age-period-cohort (APC) frameworks, which would discern three types of time-varying phenomena from the temporal trends: Age, period, and cohort effects. In this study, we used APC frameworks to investigate the long-term trend in the incidence and DALYs rates of schizophrenia in China and explored the potential effect of age, period, and cohort on the incidence and DALYs rates of schizophrenia, which would provide more information to help us understand the temporal trends of schizophrenia in China. The results are a necessary supplement to the existing research on the burden of schizophrenia in China and provide a scientific basis for evidence-based public health policies and the optimal allocation of health resources.

## Materials and Methods

### Data Sources

The data were extracted from the Global Burden of Disease (GBD) 2019 database. GBD 2019 provided a standardized, replicable approach and comprehensive estimation of incidence, prevalence, and years lived with disability for a total of 369 diseases and injuries for 204 countries and territories (13). In the GBD, the incidence data of schizophrenia in China was systematically reviewed from community representative epidemiological studies (14). Studies found were evaluated against a series of inclusion criteria, including cross-sectional or longitudinal design, reported estimates of incidence, utilized the International Classification of Diseases (ICD) or Diagnostic and Statistical Manual of Mental Disorders (DSM) diagnostic criteria, reported estimates of incidence in the form of hazard rates, representative study design, etc. More information on the inclusion criteria is available elsewhere (8). Reported estimates of incidence were entered in DisMod-MR. DisMod-MR is a Bayesian meta-regression instrument, which can compute the age-specific and sex-specific estimates from the available data (14). DALYs is a summary measure of population health, which was composed of years lived with disability and years of life lost due to premature mortality. In GBD 2019, disability weight (0.778 for acute state, and 0.588 for residual state) was used to estimate the DALYs of schizophrenia. More information on the calculation of DALYs is available elsewhere (13). The GBD world population standard was used for the calculation of age-standardized rate of schizophrenia (13).

In this study, schizophrenia was confirmed based on DSM or ICD diagnostic criteria (DSM-IV-TR: 295.10-295.30, 295.60, 295.90; ICD 10: F20) (13).

### Data Analysis

This study used the APC framework to assess the incidence and DALYs rates of schizophrenia in China and to assess the potential impact of age, period, and cohort effects on these trends. The APC model was developed based on Poisson distribution, and can be generally expressed as follows (15):


(1) $$Y=\text{log}\,\left(M\right)=\mu +\alpha \,{\mathrm{Age}}_{i}+\beta \,{\mathrm{Period}}_{j}+\gamma \,{\mathrm{Cohort}}_{k}+\varepsilon$$

Where, *M* for the incidence/DALYs rate of the corresponding age group, μ for the intercept item, α, β, and γ for the age, period and cohort effect, and ε for random error. To address the problem of identification of the model parameters (perfect collinearity of the age, period, and cohort variables), weighted least squares regression was used to partition the effects of age, period, and cohort effect (12, 16).

By using the APC framework, the following parameters were evaluated: local drifts, represents annual percentage change of the expected age-specific rates over time. The longitudinal age curve represents the age effect, which indicates the risk of incidence/DALYs of schizophrenia in different age groups in the reference cohort adjusted for period effects. Period rate ratios (RR) represent period effects, indicates the risk of incidence/DALYs of schizophrenia over the years belonging to different periods. Cohort RR represent cohort effects, indicate the risks of incidence/DALYs of schizophrenia in different birth cohorts.

To conduct APC analysis, we divided the population, incidence, and DALYs data into 6 periods from 1990–1994 (median 1992) to 2015–2019 (median 2017) at 5-year intervals. We also divided the age data into 13 consecutive age groups (10–14 years old to 70–74 years old at five-year intervals). The birth cohort was divided into 18 birth cohorts from 1918–1922 to 2003–2007 at intervals of 5 years.

Estimable parameters were estimated using the American National Cancer Institute’s APC web tool (Biostatistics Branch, National Cancer Institute, Bethesda, MD, United States) (12). By default, the average age, period, and birth cohort were set as the reference group. In this study, the central age group (40–44 years), period group (2000–2004), and birth cohort (cohort 1958–1962) were set as references.

Wald chi-square tests were used to estimate the significance of estimable functions. All statistical tests were two-sided, and a *P-value* less than 0.05 was considered statistically significant.

## Results

### Trends in the Incidence and Disability-Adjusted Life Years Rate of Schizophrenia in China for the Period From 1990 to 2019

**FIGURE 1:**
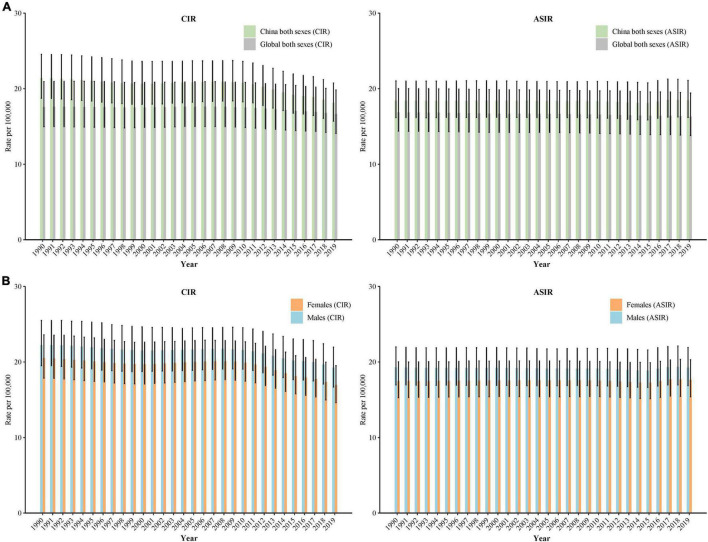
Trends in age-standardized incidence rate (ASIR) and crude incidence rate (CIR) of schizophrenia in China by sex, 1990 to 2019: **(A)** ASIR and CIR for both sexes, **(B)** ASIR and CIR for males and females. The Global Burden of Disease Study 2019 global age-standard population was used.

Figure 1A shows the age-standardized incidence rate (ASIR) and crude incidence rate (CIR) of schizophrenia in China from 1990 to 2019 in both sexes. In 1990, the ASIR of schizophrenia was 18.41/100,000, and it increased to 18.47/100,000 in 2019 (increased by of 0.3%). The CIR in 1990 was 21.40/100,000, and it decreased to 18.14/100,000 in 2019 (decreased by 15.2%).

Figure 1B shows the trends in CIR and ASIR of schizophrenia by sex from 1990 to 2019 in China. For males, the CIR decreased from 22.23/100,000 in 1990 to 19.28/100,000 in 2019 (decreased by 13.3%), and the ASIR decreased from 19.28/100,000 in 1990 to 19.26/100,000 in 2019 (decreased by 0.1%). For females, the CIR decreased from 20.51/100,000 in 1990 to 16.96/100,000 in 2019 (decreased by 17.3%), and the ASIR increased from 17.47/100,000 in 1990 to 17.66/100,000 in 2019 (increased by 1.1%).

**FIGURE 2:**
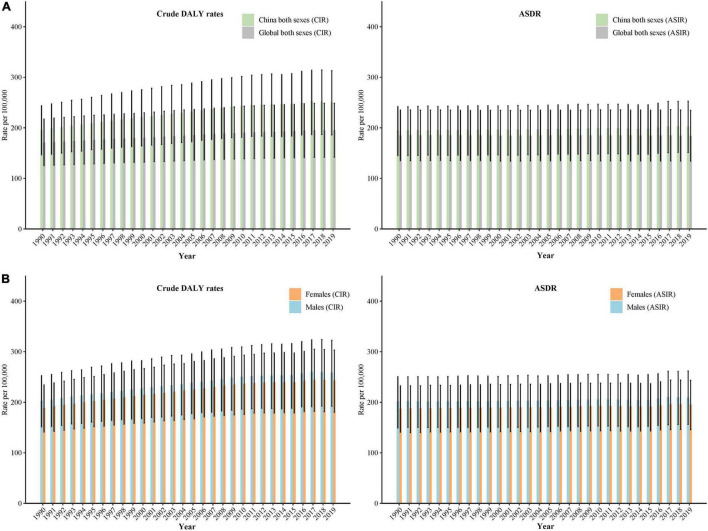
Trends in crude disability adjusted life years (DALYs) rate and age-standardized DALYs rate (ASDR) of schizophrenia in China by sex, 1990 to 2019: **(A)** ASDR and crude DALYs rate for both sexes, **(B)** ASDR and crude DALYs rate for males and females. The Global Burden of Disease Study 2019 global age-standard population was used.

In 1990, the age-standardized DALYs rate (ASDR) of schizophrenia was 195.04/100,000, and it increased to 202.42/100,000 in 2019 (increased by 3.8%). The crude DALYs of schizophrenia was 195.98/100,000, and it increased to 250.99/100,000 in 2019 (increased by 28.1%) (Figure 2A).

For males, the crude DALYs rate increased from 202.85/100,000 in 1990 to 258.54/100,000 in 2019 (increased by 27.4%), and the ASDR increased from 201.90/100,000 in 1990 to 209.30/100,000 in 2019 (increased by 3.7%). For females, the crude DALYs rate increased from 188.66/100,000 in 1990 to 243.15/100,000 in 2019 (increased by 28.9%), and the ASDR increased from 187.62/100,000 in 1990 to 195.36/100,000 in 2019 (increased by 4.1%) (Figure 2B).

### Local Drift Values for the Incidence and Disability-Adjusted Life Years Rate of Schizophrenia in China

**FIGURE 3:**
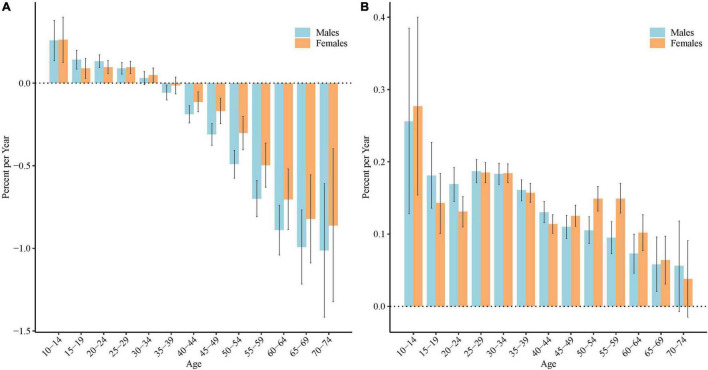
Local drift values for the incidence and disability adjusted life years (DALYs) rate of schizophrenia in China. Age group–specific annual percent change (local drift) in the incidence **(A)** and DALYs rate **(B)** of schizophrenia and corresponding 95% confidence intervals.

Figure 3 shows the annual percentage change of the expected age-specific rates (local drifts) over the last three decades. As Figure 3A shows, for males, the local drift for incidence was higher than 0 (significance with *P* < 0.05) in the age group from 10 to 29 years (local drifts, 0.01 to 0.26%), and lower than 0 (significance with *P* < 0.05) in the age group from 35 to 74 years (local drifts, −1.01 to −0.06%). For females, the local drift was higher than 0 (significance with *P* < 0.05) in the age group from 10 to 34 years (local drifts, 0.05 to 0.26%), and lower than 0 (significance with *P* < 0.05) in the age group from 40 to 74 years (local drifts, −0.86 to −0.11%). The greatest increasing of schizophrenia incidence were found in females aged 30 to 34 years (local drifts = 0.26%, 95% confidence interval [CI] = 0.13 to 0.40%). The greatest improvements was found in males aged 70–74 years (local drifts = −1.01%, 95%CI = −1.42 to −0.61%).

As Figure 3B shows, for both males and females, the local drift for DALYs was higher than 0 (significance with *P* < 0.05) in the age group from 10 to 69 years (local drifts, 0.06 to 0.26% for males and 0.06 to 0.28% for females). The greatest increasing of DALYs were found in both males and females aged 10 to 14 years, local drifts = 0.26% (95%CI: 0.13%, 0.39%) for males and 0.28% (95%CI: 0.15%, 0.40%) for females.

### Longitudinal Age Curves of the Incidence and Disability-Adjusted Life Years Rate of Schizophrenia in China

**FIGURE 4:**
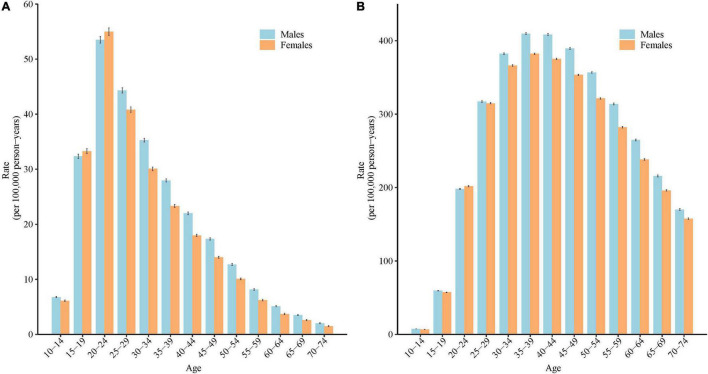
Longitudinal age curves of the incidence and disability adjusted life years (DALYs) rate of schizophrenia in China: **(A)** incidence, **(B)** DALYs rate. Fitted longitudinal age-specific rates of the incidence/DALYs rate of schizophrenia (per 100,000 person-years) and the corresponding 95% confidence intervals (some of them were too narrow to show in the figure).

Figure 4 shows the longitudinal age curve of the incidence and DALYs rate of schizophrenia. After adjusting for period effects, in the reference cohort, the incidence and DALYs rate of schizophrenia all showed a general V-shape reversal with aging, which increased firstly, then decreased. For both males and females, individuals aged 20–24 years old had the highest incidence of schizophrenia (53.48/100,000 person years for males, 54.97/100,000 person years for females), and individuals aged 35–39 years had the highest DALYs rate of schizophrenia (409.70/100,000 person years for males, 382.24/100,000 person years for females).

### Period Rate Ratios of Schizophrenia Incidence and Disability-Adjusted Life Years Rate in China

**FIGURE 5:**
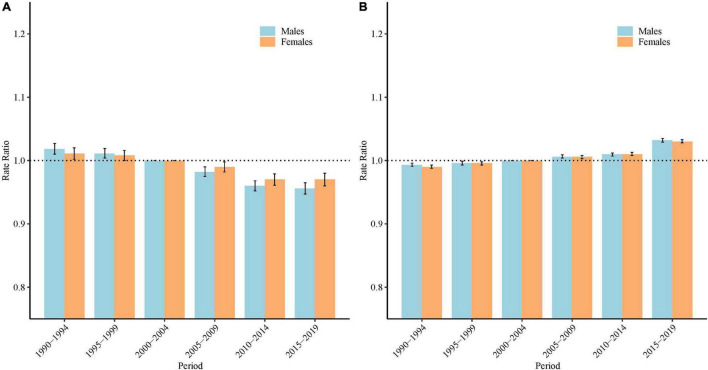
Period rate ratios (RR) of the incidence and and disability adjusted life years (DALYs) rate of schizophrenia by sex in China: **(A)** incidence, **(B)** DALYs rate. The RR of each period compared with the reference period (2000 to 2004) adjusted for age and nonlinear cohort effects and the corresponding 95% confidence intervals. The black dash lines indicates an RR = 1.

Based on the Wald tests (Table 1), the period effects were statistically significant for both sexes (*P* < 0.05 for all). Figure 5 shows the estimated period effects by sex during the whole study period. For the incidence, the period effects showed a decreasing tendency during the period from 1990–1994 to 2010–2014 for both males and females, with the period RR decreased from 1.02 to 0.96 (decreased by 5.7%), and 1.01 to 0.97 (decreased by 4.1%), respectively. However, this decreasing tendency slowed down in males (0.96 to 0.956) and stopped in females (0.97 to 0.97) in the period from 2015 to 2019. For the DALYs rate, the period RR showed similar monotonic increased patterns for both sexes (increased by 3.9% for males, and 4.0% for females).

**TABLE 1:** Wald Chi-Square tests for estimable functions in the APC model.

Null hypothesis	Incidence				DALYs rates			
	Males		Females		Males		Females	
	Chi-square	*P*-Value	Chi-square	*P*-Value	Chi-square	*P*-Value	Chi-square	*P*-Value
All period RR = 1	178.9	<0.001	67.8	<0.001	789.2	<0.001	929.7	<0.001
All cohort RR = 1	334.9	<0.001	151.0	<0.001	1070.7	<0.001	1535.2	<0.001
All local Drifts = Net Drift	334.1	<0.001	143.3	<0.001	115.8	<0.001	275.1	<0.001

*DALYs, disability-adjusted life years. RR, Rate Ratio. Net Drift, annual percentage change of the expected age-adjusted rates over time. Local Drift, annual percentage change of the expected age-specific rates over time.*

### Cohort Rate Ratios of Schizophrenia Incidence and Disability-Adjusted Life Years Rate in China

**FIGURE 6:**
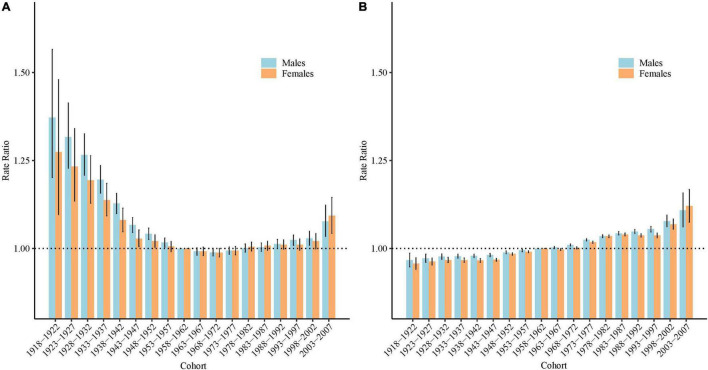
Cohort rate ratios (RR) of the incidence and disability adjusted life years (DALYs) rate of schizophrenia by sex in China: **(A)** incidence, **(B)** DALYs rate. The RR of each cohort compared with the reference cohort (1958 to 1962) adjusted for age and nonlinear period effects and the corresponding 95% confidence intervals. The black dash lines indicates an RR = 1.

Based on the Wald tests, the cohort effects were statistically significant for both sexes (*P* < 0.05 for all) (Table 1). Figure 6 shows the estimated cohort effects by sex. For the incidence, the cohort RR showed a decreasing trend for the cohort individuals born before 1972 (birth cohort 1918–1922 to 1968–1972), with cohort RR decreased from 1.37 to 0.99 (decreased by 27.9%) in males, and 1.27 to 0.99 (decreased by 22.4%) in females; however, this decreasing tendency was reversed afterward and increased sharply in the recent birth cohort (birth cohort 1968–1972 to 2003–2007), with cohort RR increased from 0.99 to 1.08 (increased by 8.9%) in males, and 0.99 to 1.08 (increased by 10.6%) in females. For the DALYs rate, the cohort RR showed general increased patterns for both sexes (increased by 14.7% for males, and 17.1% for females).

## Discussion

Our research illustrates the long-term trend in the incidence and DALYs rate of schizophrenia in China and detected the potential effects of age, period, and birth cohort. To our knowledge, this is the first study that used the APC framework to explore the trend in the incidence and DALYs rate of schizophrenia in China. Our research showed that compared with the decreased CIR, the ASIR of schizophrenia increased in China from 1990 to 2019. Although the incidence of schizophrenia decreased in the elderly population, it increased in the young population. The risk of schizophrenia incidence continued to increase in people born after 1972. For DALYs, both crude DALYs rate and ASDR increased over the past three decades, this increasing trend was found in almost all age groups, and the period and cohort effects all showed an overall increasing trend.

In our study, we found a slight increase in the ASIR of schizophrenia in China, which was not consistent with the decreasing tendency globally (7). Rapid urbanization may be the possible reason for the increase in age-standardized incidence of schizophrenia in China. Since the initiation of the reform and opening policy in 1978, China has experienced an unprecedented scale of urbanization – a nearly fivefold increase in the urban population in the past four decades (17). Urbanization creates opportunities for improved health conditions and high-quality health care, but it also brings huge health risk factors associated with schizophrenia (18), e.g., migration, crowded living conditions, stress in the working environment, and disparities in health care delivery (19), which may lead to a high risk of mental health problems for urban residents (17). In a previous study, a 10% increase in the degree of urbanicity was associated with an increased risk of schizophrenia in China (*OR* = 1.44) (17). Drug abuse may be another reason for the increased age-standardized incidence of schizophrenia in China. In the late 1980s, the drug problem reappeared in China, and spread quickly in the early 1990s (20). Previous evidence has illustrated associations between drug abuse and an increased risk of developing schizophrenia later in life (21). In addition, more willingness to admit symptoms than before (22), may also contribute to the increase of schizophrenia incidence. In this study, the crude DALYs rate and ASDR of schizophrenia in China all showed an increasing tendency. Besides the possible reason described above, increasing life expectancy in China may also play a role in increasing the burden of DALYs.

In our study, for the different sexes, females showed a slightly increasing trend of ASIR in schizophrenia. This may be related to females being more likely to be affected by the urbanization process (23). During the process of urbanization, a mass of populations leave rural areas and live and work in cities. As mentioned above, urbanization arose problems such as lack of contact with nature, social insecurity, pollution, and social disparities, which have been shown to be particularly associated with mental health among females with lower socio-economic status (24). In addition, females were more vulnerable to repeated stress exposures than males (25).

In our study, the CIR, ASIR, ASDR, and crude DALYs rate were higher in males than in females during the study period. Previous studies have reported that the incidence of schizophrenia in men is usually slightly higher than that in women, with a risk ratio of 1.4 (26). These sex differences in schizophrenia can be interpreted by different hypotheses. Hormone hypotheses emphasize that gonadal hormones played an important role in the sex differences in schizophrenia. Estrogen may play neuroprotective roles in females against schizophrenia (27). Sex chromosome hypotheses emphasize that sex chromosomes may roles in neurodevelopment and sex-specific patterns of transmission in schizophrenia patients (27). In addition, differences in brain maturity and morphology, and differences in behavior patterns for specific ages and sex may also contribute to the sex differences of schizophrenia burden (28).

In this study, we found a certain difference between the CIR (crude DALYs rate) and ASIR (ASDR) of schizophrenia for both sexes. This may be related to changes in the age structure of China. Over the past more than half a century, the proportion of young people in China has decreased, while the proportion of elderly people has increased (29). Meanwhile, schizophrenia is common among younger adults (7). Crude rates were calculated based on the whole population, which was vulnerable by the age distribution of the population. With the decline in the cases of schizophrenia, the crude incidence rate of schizophrenia has decreased. A previous study predicted that China will continue to age (30), and the reduction in the proportion of young people may further decrease the CIR of the incidence of schizophrenia. While this seems to be a good expectation in terms of the CIR of schizophrenia in China, our study also indicated that unlike the decreasing trends among the elderly, the incidence of schizophrenia in young people in China has increased in the past 30 years. It seems that the increase in the incidence of schizophrenia in young people has not reversed the downward trend in the CIR in schizophrenia in the past 30 years, but it still reminds us that more attention should be given to the problem of schizophrenia among young people in China.

Age is an important demographic risk factor that affects the incidence and DALYs of schizophrenia. In our study, we found that both the incidence and DALYs rate of schizophrenia showed a V-shape reversal with aging, which was consistent with previous studies (7). Schizophrenia nearly always occurs in late adolescence or early adulthood, when the prefrontal cortex is still developing (31). Previous longitudinal neuroimaging studies have demonstrated that the prefrontal cortex is the last to mature (32). Some researchers believe that a variety of outside factors can cause the prefrontal cortex to finish developing differently than it would have, which would cause the development of schizophrenia in young people (33). From a psychology point of view, the late-adolescent onset of schizophrenia is a consequence of blocked psychological maturation during adolescence (34), and the troubled teenage state, such like stress, depress, and limited understanding of other people’s minds, fails to be solved with normal maturation, and this state deteriorates into the appearance of schizophrenia symptoms (34).

The period effect reflects variations in the outcome over time that influence all age groups simultaneously, and the cohort effect reflects the changes in outcome across groups of individuals with the same birth year (35). Although under certain restrictions, the period effect and cohort effect can be estimated separately by the period RR and cohort RR, respectively, it is not easy to interpret them in practice (36). When the period effect influences all age groups, certain age groups would be affected simultaneously, which leads to the cohort effect to some extent. Different birth cohorts are often born in different periods, inevitably having an impact on the period effect (35). Therefore, in this study, we comprehensibly discussed the possible reasons for the trends in the period and cohort effects.

In this study, both the period effect and cohort effect for the incidence of schizophrenia show a decreasing trend in the earlier period (before 2015) and older birth cohort (those born before the year 1972). This may be related to one or more of China’s achievements in mental health. Since 1949, psychiatric hospitals have been gradually established in different provinces. Since 1958, mental health facilities were established in some provinces to train professionals and to develop work plans (early detection and treatment and relapse prevention of psychoses). Health, civil affairs, and public security departments have established a three-level network, including cities, districts/counties, and streets/towns, to prevent and treat psychoses since 1980, and successful experiences with treatment models were extended to other places. Although the demands of the economic reform that emerged over the 1990s rendered some models unviable, by the late 1990s, the Ministry of Health of China began to reconsider the principles of and approaches to mental health care. In 2002, an 8-year mental health plan was signed to build an effective mental health system, thus accelerating mental health legislation, increasing citizen awareness, strengthening mental health services, and developing mental health human resources (37). In 2004, China launched a mental health reform plan to incorporate mental health into the scope of public health (37). By 2009, the plan covered 96.88 million people in 112 cities and had trained nearly 30,000 professional and technical personnel, including psychiatrists, psychiatric nurses, community physicians, case managers, community workers, public safety personnel, and family members (38). In 2013, China’s “Mental Health Law” went into effect, which was the first national law on mental health. The government’s continued attention and investment partially explained the continued decrease in period and cohort risk of schizophrenia incidence. However, although the period effect and cohort effect show a decreasing trend in the earlier period and birth cohort, as mentioned before, the burden of schizophrenia risk factors, including urbanization, is increasing in China. The period and cohort effect already showed the potential unfavorable trends - the decreasing trend of period RR slowed down and stalled in the recent period year, and the decreasing trend of cohort RR was reversed with people born after 1972. In addition, period and cohort effects for the DALYs rate of schizophrenia all showed an unfavorable trend. As the incidence risk increases in newly born young people, schizophrenia may bring a huge burden to population health in China, which calls for more effective efforts.

For the potential increased risk of schizophrenia, more prevention and management measures are needed. The government is suggested to allocate sufficient human and facilities resources, strengthen the ability of the general practitioners to identify high-risk populations, and take early intervention to prevent high-risk individuals from developing schizophrenia (39). In addition, social support is also important for individuals to manage stressful life events and prevent triggers for schizophrenia.

Although this study used the APC framework to illustrate the long-term trend in the incidence and DALYs rate of schizophrenia in China and detect the underlying effect of this trend, which may provide supplementary information for understanding the burden of schizophrenia in China, there are still some limitations that should be noted. First, this is an ecological study; ecological studies examine the characteristics of population groups rather than individuals, and further individual-based studies are needed to confirm the results. Second, because GBD data do not distinguish the incidence/DALYs rate of schizophrenia in urban and rural areas, this study does not analyze the long-term trend of schizophrenia incidence and DALYs rate in urban and rural areas in China. Considering the difference between the prevalence of schizophrenia in this region (11), future analyses of the incidence and DALYs of schizophrenia in rural and urban areas in China are needed.

## Conclusion

In conclusion, our research shows that although the CIR of schizophrenia generally decreased in China over the last 30 years, the ASIR, ASDR, and crude DALYs rate increased. By using the APC framework, we affirmed that the incidence of schizophrenia decreased in the elderly population, but it has increased among young people, and the DALYs rate increased in almost all age groups. In the same birth cohort, the incidence and DALYs rate of schizophrenia all showed a V-shape reversal with aging, individuals aged 20–24 years old had the highest incidence of schizophrenia, and individuals aged 35–39 years old had the highest DALYs rate of schizophrenia. The estimated period and cohort RR for DALYs rate all showed potential unfavorable trends. For incidence, the decreasing trend of period RR slowed down and stalled in the recent period year, and the cohort RR increased as the birth cohort moved forward starting with those born in 1972. These potential trends all play a warning call for more effective efforts. Considering that there is no evidence that China’s urbanization process will stop soon, it is necessary to pay attention to vulnerable populations, and policies also need to be formulated to promote mental health.

## Data Availability Statement

Publicly available datasets were analyzed in this study. This data can be found here: http://ghdx.healthdata.org/gbd-2019.

## Author Contributions

WD and RB initially conceived the research idea, designed the study, performed data collection, management, and analysis. ZS provided administrative support. WD and RB drafted the original manuscript. YuL, JS, YaL, and ZS critically revised the manuscript. All co-authors have read and approved the final manuscript.

## Conflict of Interest

The authors declare that the research was conducted in the absence of any commercial or financial relationships that could be construed as a potential conflict of interest.

## Publisher’s Note

All claims expressed in this article are solely those of the authors and do not necessarily represent those of their affiliated organizations, or those of the publisher, the editors and the reviewers. Any product that may be evaluated in this article, or claim that may be made by its manufacturer, is not guaranteed or endorsed by the publisher.
